# The Canadian experience using the expanded criteria donor classification for allocating deceased donor kidneys for transplantation

**DOI:** 10.1186/s40697-016-0106-9

**Published:** 2016-03-24

**Authors:** Ann Young, Stephanie N. Dixon, Greg A. Knoll, Amit X. Garg, Charmaine E. Lok, Ngan N. Lam, S. Joseph Kim

**Affiliations:** Department of Medicine, University of Toronto, Toronto, Ontario Canada; Institute for Clinical Evaluative Sciences, Ontario, Canada; Division of Nephrology, University of Ottawa, Ottawa, Ontario Canada; Division of Nephrology, Western University, London, Ontario Canada; Division of Nephrology, University of Alberta, Edmonton, Alberta Canada; Division of Nephrology, Toronto General Hospital, University Health Network, University of 585 University Avenue, 11-PMB-129 Toronto, Ontario Canada

## Abstract

**Background:**

Although the outcomes of transplantation with expanded criteria donor (ECD) kidneys are inferior to non-ECD transplants in the USA, the impact of the ECD classification on Canadian kidney transplant recipients is not known.

**Objectives:**

The objective of the study was to assess the performance of the US-derived ECD classification among deceased donor kidney transplant recipients in a Canadian setting.

**Design:**

This study was a population-based cohort study.

**Setting:**

The study was conducted in all adult kidney transplant centers in the province of Ontario.

**Patients:**

The patients were incident-deceased donor kidney transplant recipients from January 1, 2005 to March 31, 2011.

**Measurements:**

Study subjects were identified through the Trillium Gift of Life Network and linked to healthcare databases in Ontario. ECD status was based on age, hypertension, kidney function, and stroke-related death. Outcomes of interest included graft loss, death, and delayed graft function.

**Methods:**

The Kaplan-Meier product limit method was used to graphically assess time to graft loss or death. Multivariable Cox proportional hazards models were used to assess graft loss or death as a function of ECD status. Multivariable logistic regression models were fitted for the outcome of delayed graft function.

**Results:**

Of 1422 deceased donor kidney transplants, 325 (23 %) were from ECDs. The median donor age was 63 vs. 42 years for ECD vs. non-ECD, respectively. The 5-year cumulative incidence of total graft loss was 29.2 % in ECD and 20.7 % in non-ECD kidney transplants. The relative hazards for total graft loss (HR 1.48 [95 % CI, 1.10; 2.00]) and death-censored graft loss (HR 1.80 [95 % CI, 1.19, 2.71]) were increased in ECD vs. non-ECD transplants. Increased relative risks were also observed for death and delayed graft function, albeit not statistically significant.

**Limitations:**

Although comprehensive in coverage and outcome ascertainment, the available details on covariate data may be limited in large healthcare databases.

**Conclusions:**

The ECD classification identifies kidneys at increased risk for graft loss in Canadian patients. The performance of more granular measures of donor risk (e.g., Kidney Donor Risk Index) and its impact on organ allocation/utilization in Canadian patients requires further study.

**Electronic supplementary material:**

The online version of this article (doi:10.1186/s40697-016-0106-9) contains supplementary material, which is available to authorized users.

## Abrégé

**Mise en contexte:**

Aux États-Unis, le classement des donneurs répondant à des critères élargis (classement DCE) a déjà permis d’établir que l’issue favorable d’une greffe de rein provenant d’un tel donneur était inférieure aux résultats obtenus avec des reins provenant de donneurs ne faisant pas partie de ce classement. Toutefois, nous ne connaissons toujours pas les répercussions d’un tel classement sur les receveurs d’une greffe de rein au Canada.

**Objectifs de l’étude:**

L’étude visait à évaluer, dans le contexte canadien, l’efficacité de la mise en application du classement DCE américain pour les receveurs de greffe du rein provenant de donneurs décédés.

**Cadre et type d’étude:**

L’étude de type cohorte représentative de la population s’est tenue au sein de tous les centres de transplantation rénale pour adultes de l’Ontario.

**Patients:**

L’étude s’est tenue en répertoriant tous les patients qui ont reçu une greffe de rein provenant de donneurs décédés d’un accident vasculaire cérébral (AVC) entre le 1er janvier 2005 et le 31 mars 2011.

**Mesures:**

Les sujets susceptibles de pouvoir participer à l’étude ont été repérés grâce au *Réseau Trillium pour le don de vie* et reliés aux bases de données du système de santé de l’Ontario. Le statut du donneur au classement DCE a été établi en fonction de son âge, de la présence ou non d’un historique d’hypertension artérielle, de sa fonction rénale et du fait que la mort cérébrale soit conséquente d’un AVC. Les événements indésirables retenus pour le suivi incluaient la perte du greffon, le décès du receveur ou un retard dans la reprise de la fonction du greffon après la transplantation.

**Méthodologie:**

La méthode de Kaplan-Meier a été employée pour représenter graphiquement le temps entre la transplantation et la perte du greffon ou le décès. Le modèle de régression de Cox à variables multiples a été utilisé pour évaluer ces deux mêmes critères en fonction du statut du donneur au classement DCE. Enfin, on a eu recours à des modèles de régression logistique à variables multiples pour ajuster les résultats dans les cas de retard de la fonction du greffon après l’intervention chirurgicale.

**Résultats:**

Parmi les 1422 donneurs décédés répertoriés pour la période couverte par l’étude, on a dénombré 325 (23 %) donneurs répondant à des critères élargis (DCE). Leur âge médian se situait à 63 ans contre 42 ans dans le cas des donneurs non classés DCE. L’incidence cumulative de la perte totale du greffon après cinq ans était de 29,2 % pour les reins provenant de DCE alors qu’elle n’était que de 20,7 % pour les reins de donneurs non classés DCE. Le risque relatif (RR) de perte totale du greffon chez les receveurs survivants (RR 1,48 [95 % IC : 1,10, 2,00]) et chez les receveurs décédés en cours d’étude (RR 1,80 [95 % IC : 1,19, 2,71]) était plus élévé lorsque le greffon provenait de DCE que de donneurs non classés DCE. On a également noté une augmentation du risque relatif de décès du receveur ainsi que du retard de reprise de fonction du greffon, quoique ces données n’aient pas atteint un degré acceptable de signification statistique.

**Limites de l’étude:**

Malgré le fait que l’étude soit complète en ce qui concerne la couverture des événements indésirables et des résultats obtenus, il reste que les détails relatifs aux données covariables sont parfois limités dans les grandes bases de données des systèmes de santé.

**Conclusion:**

L’étude a démontré que le classement DCE permettait de recenser les reins disponibles pour transplantation présentant un risque élevé de perte du greffon chez les patients canadiens. Une analyse plus approfondie présentant une meilleure distribution statistique des risques pour le donneur en se référant notamment au *Kidney Donor Risk Index* (KDRI), et de leurs répercussions engendrées sur l’attribution ou l’utilisation des organes chez les patients canadiens s’avère essentielle.

## What was known before

The expanded criteria donor (ECD) classification system was originally derived in a US cohort of to assess the impact of deceased donor kidney quality on transplant outcomes. It has been implemented in allocation algorithms, not only in the USA but also in other parts of the world. However, there is a paucity of validation studies evaluating the usefulness of this classification system in non-US settings.

## What this adds

The ECD classification system is predictive of a group of deceased donor kidneys at increased risk of long-term graft failure in a Canadian context. However, there does not appear to be any additional predictive value of the ECD system beyond donor age alone in this study population. The performance of more granular measures of deceased donor kidney quality in Canadian patients, such as the Kidney Donor Risk Index, requires future study.

## Introduction

Due to the demand for life-saving kidneys for the treatment of end-stage renal disease (ESRD), transplant programs now accept organs that may have a higher risk of complications. In 2002, the US-derived classification for ECD was formally introduced [1]. The United Network for Organ Sharing (UNOS) defined ECD as “any brain dead donor ≥60 years, or 50 to 59 years with two or more of: a history of hypertension, pre-terminal serum creatinine ≥1.5 mg/dL (133 μmol/L), or death due to stroke” [2]. Having these factors increase graft failure risk by at least 70 % among US kidney transplant recipients [1].

By 2006, most Canadian organ procurement organizations, including Ontario’s Trillium Gift of Life Network (TGLN), started using the ECD classification to allocate deceased donor kidneys [3]. There are inherent differences in patient case mix (e.g., fewer African-American patients in Canada; more metabolic syndrome in the USA), provision of health services (e.g., post-transplant surveillance; government funding for a portion of the costs of immunosuppression), and outcomes between Canadian and US kidney transplant recipients [4–9]. Given these differences, whether the ECD classification can risk-stratify Canadian deceased donor kidney transplants remains a question. This study assessed the performance of the US-derived ECD classification among deceased donor kidneys in a Canadian setting.

## Methods

### Design and setting

This was a retrospective, population-based, cohort study of deceased donor kidney transplant recipients in Ontario, Canada from January 1, 2005 to March 31, 2011. Ontario residents have universal access to hospital care and physician services, allowing for data linkage and long-term follow-up through provincial health administrative datasets. This study was approved by the institutional review boards at the University Health Network and Sunnybrook Health Sciences Centre, Toronto, Canada.

### Data sources

Data from deceased donors were abstracted from medical records housed at TGLN [10]. Provincial data at the Institute for Clinical Evaluative Sciences (ICES) used to ascertain recipient characteristics and outcomes included: (1) the Canadian Organ Replacement Register; (2) diagnostic and procedural information from hospital admissions, emergency room visits, and outpatient encounters from the Canadian Institute for Health Information data sources; (3) physician billing claims from the Ontario Health Insurance Plan; (4) demographic and vital status from Ontario’s Registered Persons Database; and (5) diabetes and hypertension data from ICES-derived cohorts. These datasets were linked using unique encoded identifiers and analyzed at the Institute for Clinical Evaluative Sciences (ICES).

### Study population

The study included all consecutive recipients of first deceased donor kidney transplants in Ontario, Canada during the cohort accrual period. Kidney transplants involving donors and/or recipients <18 years of age, living donor kidney transplant recipients, dual kidney transplants, multi-organ transplants, and transplants using out-of-province deceased donor kidneys were excluded.

### Exposure assessment

Recipients were dichotomized based on ECD status. The UNOS definition was modified such that kidney function was defined using a Modification of Diet in Renal Disease (MDRD) estimated glomerular filtration rate (eGFR) of ≤70 mL/min/1.73 m^2^ instead of pre-terminal serum creatinine ≥1.5 mg/dL. This reflected how the TGLN algorithm defined ECD kidneys and how they were actually allocated in Ontario [2, 11]. All components of the ECD criteria were verified through manual data abstraction. Baseline characteristics were ascertained using data from TGLN and the Canadian Organ Replacement Register.

### Transplant outcomes

All recipients were followed from their transplant date. The primary outcome was total graft loss, which considered time from transplantation to a composite of return to chronic dialysis, pre-emptive re-transplant, or death with graft function. Other outcomes included delayed graft function, death-censored graft loss, death with graft function, and all-cause mortality. Graft loss was defined based on having healthcare codes for chronic dialysis separated by at least 90 days (but less than 150 days) or re-transplantation as indicated by TGLN data [12]. Delayed graft function was defined by at least one healthcare code for dialysis within 7 days post-transplantation. Recipients were censored if they emigrated from the province during the study period or reached the end of the study (March 31, 2012).

### Statistical analysis

Baseline characteristics were reported by donor ECD status and compared using *t* tests or chi-square tests. ECD status was entered as a dichotomous variable in statistical models. Modeling strategies varied by outcome. For delayed graft function, multivariable logistic regression analyses were performed, and odds ratios (OR) with 95 % confidence intervals (CI) were reported. For total graft loss, Cox proportional hazards models were used. To assess each component of the composite outcome, Cox proportional hazards models were used to model the cause-specific hazard, censoring for the competing event (e.g., death-censored graft loss). Multivariable models were stratified by transplant center to allow the baseline hazard vary by site. Clinically relevant potential confounders included recipient age, sex, race, cause of ESRD, panel reactive antibody level, pre-transplant dialysis time, re-graft, and the Charlson comorbidity index. Donor characteristics included sex, race, body mass index, history of other medical conditions (e.g., diabetes, hyperlipidemia, proteinuria), and whether the donation was after cardiac death. Transplant characteristics included human leukocyte antigen mismatch, use of pulsatile perfusion, cold ischemia time, and year of transplantation. Hazard ratios (HR) with the 95 % CIs were reported.

The proportional hazards assumption was assessed by the correlation between the Schoenfeld residuals and ECD status. It was considered appropriate if the test for correlation was >0.05. Exploratory, subgroup analyses assessed the risk of total graft loss by factors that impacted the benefit of being listed for an ECD kidney in other populations [13]. These included recipient diabetes as a comorbidity (Y/N), recipient age (≥ or <40 years), duration of dialysis (≥ or <4 years), and transplant date (pre- or post-2008). Sensitivity analyses fitted proportional hazards models for the sub-distribution hazard as described by Fine and Gray [14, 15]. This assessed the effect of ECD status on the cumulative incidence of each event of interest (e.g., graft loss) while accounting for other events (e.g., death) as competing events. The impact on outcomes with donors dichotomized according to the original UNOS ECD definition was also performed. The likelihood ratio test was used to compare models to explore the benefit of using the ECD metric above and beyond the use of done age alone. All analyses were conducting using SAS version 9.3 (SAS Institute Inc., Cary, North Carolina, USA) and R version 2.15.1 (R Foundation for Statistical Computing, Vienna, Austria).

## Results

### Baseline characteristics

**Fig. 1:**
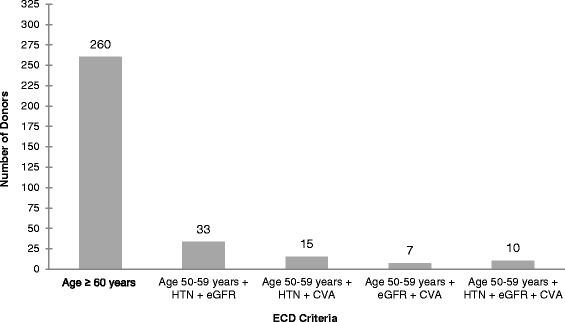
Distribution of ECD classification elements by deceased donors in the study cohort. Note: eGFR criteria ≤ 70 mL/min Abbreviations: HTN: history of hypertension; eGFR: estimated glomerular filtration rate; CVA: death due to a stroke.

This study followed 1422 deceased donor kidney transplant recipients across all six adult transplant hospitals in Ontario, Canada from January 1, 2005 to March 31, 2011. The median follow-up time was 3.1 years (4689 person-years of follow-up). Overall, 325 (23 %) of the transplanted kidneys were ECD and 1097 were non-ECD. The proportion of ECD kidneys was between 21 and 24 % from 2005 to 2010. The majority (80 %) of ECD donors were classified as expanded criteria based on their age at the time of death (Fig. 1). The next largest contribution was from donors aged 50 to 59 years, who had a history of hypertension and reduced eGFR (10 %). This ECD criteria distribution was consistent across individual centers.

Kidney transplant recipient characteristics by ECD status are shown in Table 1. When comparing recipients of ECD kidneys to non-ECD kidneys, the former was older (median age, 62 vs. 54 years), mostly Caucasian (62 %), and had a variety of ESRD causes. Diabetes and cardiovascular disease were more prevalent amount recipients of ECD kidneys compared to non-ECD kidney recipients (44 vs. 32 % for diabetes and 11 vs. 7 % for cardiovascular disease). Recipients of ECD kidneys spent about 5 months less on dialysis prior to transplant (54 vs. 59 months, *p* = 0.08) compared to recipients of non-ECD kidneys.

**Table 1:** Kidney transplant recipient characteristics by ECD status

	Total *N* = 1422	ECD *n* = 325	Non-ECD *n* = 1097	*p* value
Age at transplant, years				
Mean (SD)	54 (13)	59 (11)	53 (13)	<0.001
Women	503 (35 %)	105 (32 %)	398 (36 %)	0.188
Race				
Caucasian (%)	884 (62 %)	197 (61 %)	687 (63 %)	0.777
Asian (%)	121 (9 %)	32 (10 %)	89 (8 %)	
African-American (%)	127 (9 %)	31 (10 %)	96 (9 %)	
Aboriginal (%)	36 (3 %)	6 (2 %)	30 (3 %)	
Other (%)	236 (17 %)	56 (17 %)	180 (16 %)	
Unknown	18	–	–	
Primary cause of ESRD				
Glomerulonephritis (%)	328 (29 %)	68 (26 %)	260 (31 %)	0.010
Diabetes mellitus (%)	215 (19 %)	66 (25 %)	149 (18 %)	
Hypertension (%)	95 (9 %)	30 (11 %)	65 (8 %)	
Other (%)	474 (43 %)	97 (37 %)	377 (44 %)	
Unknown	310	–	–	
Pre-transplant dialysis time (months)				
Median (Q1–Q3)	58 (34-85)	54 (36–76)	59 (33–86)	0.075
Peak PRA >0 % (%)	792 (56 %)	186 (57 %)	606 (55 %)	0.134
Missing	122	–	–	
Re-graft (%)^a^	96 (7 %)	8 (2 %)	88 (8 %)	<0.001
Comorbidities				
Diabetes mellitus (%)	491 (35 %)	144 (44 %)	347 (32 %)	<0.001
Hypertension (%)	1334 (94 %)	310 (95 %)	1024 (93 %)	0.198
Cardiovascular disease (%)	117 (8 %)	36 (11 %)	81 (7 %)	0.033
Mean Charlson comorbidity index (SD)^b^	2.6 (1.4)	2.7 (1.5)	2.5 (1.4)	0.03

Reported values in percent are based on a denominator excluding unknown values.

^a^Recipients of failed living donor kidney transplants now receiving first time deceased kidney transplant.

^b^The Charlson score is a validated index of comorbidity [33].

*ESRD* end-stage renal disease, *PRA* panel reactive antibody

Donor characteristics are shown in Table 2. Kidneys from ECD were older (median age 63 vs. 42 years), had a higher prevalence of hypertension (62 vs. 22 %), death due to stroke (22 vs. 9 %), and lower eGFR (median 84 vs. 102 mL/min/1.73 m^2^) compared to their non-ECD counterparts. They were also more likely to have a higher BMI and diabetes mellitus. Donation after circulatory death was lower in the ECD group (7 vs. 17 %). Pulsatile perfusion pumps were more commonly used in the ECD group (34 vs. 15 %). A summary of transplant outcomes by ECD status is shown in Additional file 1: Table S1.

**Table 2:** Donor and transplant characteristics by ECD status

	Total *N* = 1422	ECD *n* = 325	Non-ECD *n* = 1097	*p* value
Donor characteristics				
Age at time of death, years				
Mean (SD)	47 (14)	63 (6)	42 (12)	<0.001
<50 years	729 (51 %)	0	729 (66 %)	<0.001
50 to 59 years	433 (30 %)	65 (20 %)	368 (34 %)	
≥60 years	260 (18 %)	260 (80 %)	0	
Women	616 (43 %)	152 (47 %)	464 (42 %)	0.153
Body mass index (kg/m^2^)				
Mean (SD)	27.2 (5.7)	28.5 (4.9)	26.8 (5.9)	<0.001
Pre-terminal serum creatinine, μmol/L				
Median (Q1–Q3)	66 (53-83)	68 (54-87)	66 (53-82)	<0.001
Pre-terminal eGFR, mL/min				
Median (Q1–Q3)	99 (77–127)	84 (68–110)	102 (81–130)	<0.001
Cause of death				
Cerebrovascular accident	172 (12 %)	71 (22 %)	101 (9 %)	<0.001
Trauma	119 (8 %)	23 (7 %)	96 (9 %)	
Other	1131 (80 %)	231 (71 %)	900 (82 %)	
Past medical history				
Diabetes mellitus	147 (10 %)	68 (21 %)	87 (8 %)	<0.001
Hyperlipidemia	33 (2 %)	11 (3 %)	26 (2 %)	0.147
Hypertension	518 (36 %)	223 (69 %)	295 (27 %)	<0.001
Donation after circulatory death	205 (14)	22 (7 %)	183 (17 %)	<0.001
Transplant Characteristics				
Year of transplant				
2005	150 (11 %)	33 (10 %)	117 (11 %)	0.414
2006	208 (15 %)	44 (14 %)	164 (15 %)	
2007	241 (17 %)	54 (17 %)	187 (17 %)	
2008	213 (15 %)	52 (16 %)	161 (15 %)	
2009	278 (20 %)	64 (20 %)	214 (20 %)	
2010	266 (19 %)	57 (18 %)	209 (19 %)	
2011 (until March 31)	66 (5 %)	21 (6 %)	45 (4 %)	
HLA mismatch (max 6)				
0	16 (1 %)	≤5	16 (1 %)	0.269
1	32 (2 %)	9 (3 %)	23 (2 %)	
2	62 (4 %)	17 (5 %)	45 (4 %)	
3	127 (9 %)	28 (9 %)	99 (9 %)	
4	300 (21 %)	61 (19 %)	239 (22 %)	
5	598 (42 %)	135 (42 %)	463 (42 %)	
6	287 (20 %)	75 (23 %)	212 (19 %)	
Pulsatile perfusion pump use	275 (19 %)	111 (34 %)	164 (15 %)	<0.001
Cold ischemia time, minutes				
Median (Q1–Q3)	691 (579–813)	691 (574–772)	691 (580–829)	0.098

Cells with ≤5 observations were suppressed to prevent indirect identification of individuals

*HLA* human leukocyte antigen

### Primary outcome: total graft loss

**Fig. 2:**
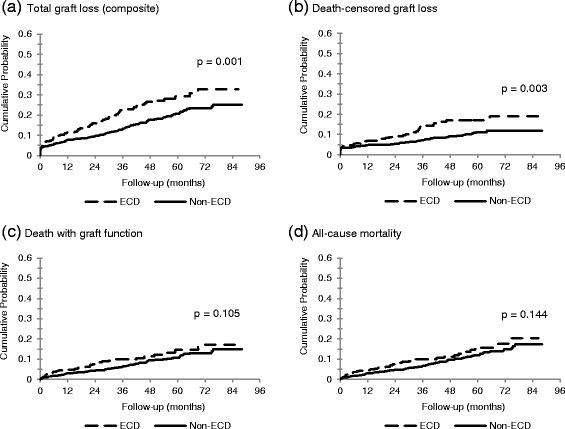
Kaplan-Meier event curves according to study group for **a** total graft loss (composite), **b** death-censored graft loss, **c** death with graft function, and **d** all-cause mortality. Note: *P*-values from a log rank statistic to compare the strata

**Fig. 3:**
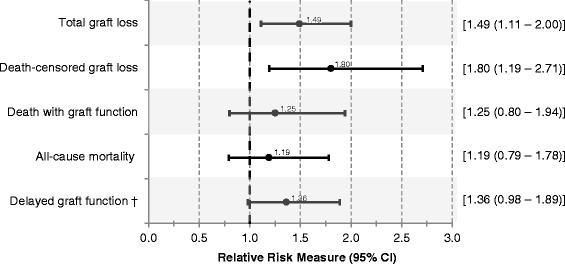
Adjusted relative risks for study endpoints comparing recipients of ECD to non-ECD kidneys. †All results reported as hazard ratios (95 % CI), except delayed graft function - reported as odds ratio (95 % CI). Models were adjusted for various donor, recipient and transplant characteristics. Please see ‘Materials and Methods’ for further details

There were 255 total graft loss events. Comparing the ECD to the non-ECD group, the cumulative probability of total graft loss was 11.4 vs. 7.7 % at 1 year, 22.8 vs. 13.2 % at 3 years and 29.2 vs. 20.7 % at 5 years (Fig. 2a). A 20 % failure rate was observed at 2.8 years for ECD donors vs. 4.8 years for non-ECD donors. Figure 3 shows relative risks comparing ECD to non-ECD kidneys. The adjusted relative hazard for total graft loss for recipients of ECD kidneys was significantly increased (HR 1.49 [95 % CI, 1.11, 2.00]).

### Death-censored graft loss

There were 134 occurrences of death-censored graft loss. The absolute risk at 1, 3, and 5 years was 6.9, 14.3, and 17.0 % for recipients of ECD kidneys, respectively. This was higher than the absolute risks of 4.9, 7.1, and 11.1 % for non-ECD kidney recipient at the same time points (Fig. 2b). The adjusted HR for death-censored graft loss was 1.80 (95 % CI, 1.19, 2.71) (Fig. 3).

### Recipient death

There were 141 deaths. At 1, 3, and 5 years, the absolute risks for death with graft function were 4.6, 10.0, and 15.7 % in the ECD group (Fig. 2c). For the non-ECD group, absolute risks were lower at 3.0, 6.8, and 11.9 % (Fig. 2c). There was no statistically significant difference in the adjusted relative hazard of death with graft function by ECD status (HR 1.25 [95 % CI, 0.80, 1.94]). Similar estimates were observed for all-cause mortality.

### Delayed graft function

The incidence of delayed graft function among ECD vs. non-ECD kidney transplant recipients was 31 vs. 24 %, respectively (Table 3). The adjusted odds ratio for delayed graft function was increased, but not statistically significant (OR 1.36 [95 % CI, 0.98, 1.89]) (Fig. 3).

**Table 3:** Assessing the ECD metric compared to donor age alone

Model	−2 Log likelihood	*df*	Likelihood-ratio statistic	*df*	*p* value
Donor age	2573	23	0.30	1	0.59
Donor age + ECD	2572	24			
ECD	2578	23	6.09	1	0.01
ECD + donor age	2572	24			
Donor age ≥60	2573	24	4.18	4	0.38
Donor age ≥60 + other ECD components	2569	28			

*ECD* expanded criteria donor (dichotomous variable), *df* degrees of freedom

### Subgroup analyses

**Fig. 4:**
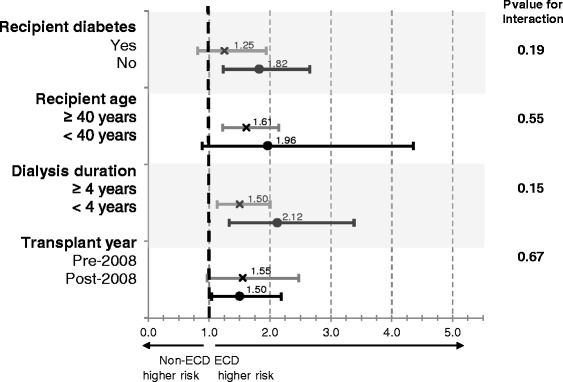
Adjusted relative risk of total graft loss comparing recipients of ECD to non-ECD kidneys by pre-defined recipient subgroups

Subgroup analyses considering the outcome of total graft loss, and comparing ECD to non-ECD kidney recipients, are summarized in Fig. 4. Overall, ECD (vs. non-ECD) kidney transplants were associated with an increased risk for total graft loss across all subgroups analyzed (*p* values for interaction = 0.15 to 0.67).

### Sensitivity analyses

Subdistribution HR estimates for graft loss accounting for death as a competing risk and death with graft loss as a competing risk were similar to the main analysis.

Applying the UNOS ECD definition for kidney dysfunction (i.e., pre-terminal serum creatinine ≥1.5 mg/dL or 133 μmol/L) to our cohort led to 286 recipients identified as receiving ECD kidneys and 1136 receiving non-ECD kidneys. Fewer donors met the kidney function criteria, resulting in only 26 donors between 50 and 59 years classified as ECD (i.e., 9 % vs. 20 % in the main analyses). Overall, there was no change in the observed outcomes. There remained a statistically significant increase in the adjusted relative hazard for total graft loss (HR 1.42 [95 % CI, 1.04, 1.94]) and a non-significant increase in the adjusted relative hazard for death with graft function (HR 1.29 [95 % CI, 0.82, 2.01]) in ECD vs. non-ECD kidney recipients.

Model comparisons to assess the use of the ECD metric compared to donor age alone are shown in Table 3. The addition of the ECD binary indicator did not appear to add additional information above and beyond a model with donor age alone (*p* = 0.59). However, donor age as a continuous indicator seemed to provide prognostic information in addition to ECD (*p* = 0.01). Separating the components of the ECD indicator, adding the criteria beyond donor age >60 years was not statistically warranted in comparison to the model with donor age > 60 years alone (*p* = 0.38).

## Discussion

In Canada, kidneys from deceased donors made up approximately 60 % of the donor pool over the last decade [5]. The quality of these kidneys varied substantially. Since the outcomes of Canadian vs. US kidney transplant recipients have been shown to differ [16], and the ECD classification was derived in a US cohort [17], a formal evaluation of its performance in a non-US setting was felt to be important. To our knowledge, this is the first assessment of the ECD classification system in a Canadian kidney transplant population. We found that for Canadian recipients of ECD vs. non-ECD kidneys, the relative hazard was significantly increased for total graft loss (by 1.5-fold) and for death-censored graft loss (by 1.8-fold). Recipients of ECD kidneys did not appear to be at a significantly higher risk for death compared to their non-ECD kidney counterparts. Based on our findings, the ECD classification system is able to identify donor kidneys at higher risk of graft loss in a Canadian setting; however, ECD as a construct for “higher risk” may not add more above and beyond older age.

In 2006, a national forum on kidney allocation in Canada was held which explicitly called for a validation of the UNOS definition in a Canadian setting, as well as periodic re-evaluation [3]. This study responded to this call and provides some support after almost a decade since implementation. The results herein are consistent with a previous systematic review of ECD kidney transplant outcomes with 28 US-registry studies reporting significantly worse 1- to 15-year patient and graft survival for ECD kidney recipients [13]. In non-US settings, a retrospective cohort study of the Australia and New Zealand Dialysis and Transplant Registry (ANZDATA) showed similarly poor outcomes [18]. More recently, a prospective study from France further corroborated these results in their population [19]. Despite this, studies have shown that these ECD recipients still have reduced mortality compared to waiting on dialysis for a standard criteria donor [20–23].

In Ontario, Canada, a different kidney function criteria was used compared to the UNOS-based ECD definition, where instead of a serum creatinine cutoff of ≥1.5 mg/dL (133 μmol/L), an MDRD eGFR cutoff of ≤70 mL/min [24] was used. In our cohort, the ECD definition using the eGFR cutoff conservatively classified more donors as ECD status (an additional 39 ECD). Re-analyzing the data as per the UNOS definition, the relative hazard for total graft loss remained significantly higher for ECD kidney transplants (HR 1.42 vs. 1.48 in the primary analysis). Similarly, an increased risk for death-censored graft loss was observed. Based on this sensitivity analysis, it is unlikely that kidney function was driving the association between ECD status and graft loss. As suggested by previous studies, increased terminal serum creatinine at the time of organ recovery may not be as closely correlated to long-term transplant outcomes [25, 26].

From a methodological perspective, this study also considered competing risks of graft loss or death, since treating competing events as censored observations overestimates actual risks in the presence of significant competing events [27]. Herein, the estimates from competing risks analyses were similar, with only a slight attenuation of the observed hazard ratios. Since those experiencing graft loss can return to chronic dialysis rather than progressing to death with a failed transplant, less dependence between failure types (i.e., graft loss and death) is not surprising. Results from both traditional and competing risks analyses are useful. Traditional models establish whether there are observed associations between ECD status and transplant outcomes, while the competing risks models evaluate the association (direct and indirect) in clinical practice [28].

The current allocation system in Ontario directs ECD kidneys to patients who are ≥60 years old, diabetics >50 years old, or recipients with other significant comorbidities [24]. Generally, these prognostically unfavorable characteristics portend a lower survival probability on the waiting list. [21] Exploratory subgroup analyses showed that the relative risk of graft loss remained increased but to a lesser degree for diabetic recipients, those ≥40 years old, and those on dialysis for ≥ 4 years. This suggests that in older patients with more comorbid conditions, the quality of the kidney they receive may not be as important. To know whether these patients would ultimately benefit more compared to staying on the waiting list would require a survival benefit analysis which was outside the scope of this study. Notably, transplant era did not seem to modify this effect.

When exploring the value of the ECD metric as a construct for “higher risk” compared to age alone, multiple model comparisons suggested that the ability of the ECD indicator to risk-stratify in a Canadian population was largely driven by donor age. These findings may be a function of the cohort, given that the vast majority of those flagged as ECD were classified on the basis of donor age. While Ontario has already implemented the ECD classification system in its allocation algorithm, some provinces continue to use older age cut-points in lieu of ECD for allocation. Moving forward, there would be limited value in applying the ECD classification system to these areas. Rather, the identification of prognostic factors beyond donor age specifically relevant to the Canadian transplant population would improve granularity in assessing donor quality for allocation in Canada.

The major strength of this study is the use of large healthcare databases in Ontario to address a recognized knowledge gap in the current practice of deceased donor kidney transplantation. Limitations include inadequate capture of relevant data elements, such as donor kidney biopsy data to assess kidney quality, immunosuppression and associated side effects, and acute rejection episodes [29–31]. The lack of data on discarded kidneys may have impacted the demographic breakdown of our ECD cohort (i.e., 80 % ECD kidneys were classified as such due to older age as other categories of ECD may have had a higher propensity to be discarded). This limited the ability to fully model risks in order to suggest which kidneys should have been used. As mentioned above, the current system in Ontario directs ECD kidneys to patients who are ≥60 years old, diabetics >50 years old, or recipients with other significant comorbidities. This analysis is, thus, subject to confounding by indication, despite adjustment for recipient age and comorbidities.

## Conclusions

Overall, the impact of utilizing the ECD classification to risk-stratify deceased donor kidneys extends beyond the US population; validation in non-US settings remains as an important issue. This study provides evidence that the US-derived ECD classification system has its merits when applied to a Canadian kidney transplant population. Yet its value beyond donor age as a method to risk-stratify remains questionable. Moreover, there is variability in the quality even among ECD kidneys [32]. More recently, a continuous measure of risk called the Kidney Donor Risk Index (KDRI) has been implemented in the USA to risk-stratify deceased donor kidneys [32]. It incorporates more donor factors to better capture elements of deceased donor kidney quality. Canada has not yet followed suit with the implementation of this system. The performance of the KDRI or more granular measures of donor risk specific to Canadian patients requires further study.

## Additional file

Additional file 1: Table S1. Summary of transplant outcomes by ECD status. (DOCX 38 kb)

## Abbreviations

CI: confidence interval
ECD: expanded criteria donor
eGFR: estimated glomerular filtration rate
HR: hazard ratio
ICES: Institute for Clinical Evaluative Sciences
MDRD: modification of diet in renal disease
OR: odds ratio
TGLN: Trillium Gift-of-Life Network
UNOS: United Network for Organ Sharing
