# Supplementary material for: The Canadian experience using the expanded criteria donor classification for allocating deceased donor kidneys for transplantation
Source: Can J Kidney Health Dis. 2016 Mar 24;3:15. doi: 10.1186/s40697-016-0106-9 (PMC4806479; doi:10.1186/s40697-016-0106-9)
Supplement: Additional file 1: Table S1. — Summary of transplant outcomes by ECD status. (DOCX 38 kb) [file 40697_2016_106_MOESM1_ESM.docx]

**SUPPLEMENTARY TABLE 1.** Summary of transplant outcomes by ECD status

|  | **Total**  **N = 1422** | **ECD**  **N = 325** | **Non-ECD**  **N = 1097** |
| --- | --- | --- | --- |
| Delayed graft function |  |  |  |
| Number of events (%) | 367 (26%) | 95 (31%) | 272 (24%) |
| Total follow-up time, person-years | 4689 | 991 | 3699 |
| Total graft loss (graft failure or all-cause death) |  |  |  |
| Number of events (%) | 255 (18%) | 76 (23%) | 179 (16%) |
| Events per 1,000 patient years | 54.4 | 76.7 | 48.4 |
| Graft loss (return to dialysis or re-transplant) |  |  |  |
| Number of events (%) | 134 (9%) | 43 (13%) | 91 (8%) |
| Events per 1,000 patient years | 28.6 | 43.4 | 24.6 |
| Death with functioning graft |  |  |  |
| Number of events (%) | 121 (9%) | 33 (10%) | 88 (8%) |
| Events per 1,000 patient years | 25.8 | 33.3 | 23.8 |
| All-cause death |  |  |  |
| Total follow-up time, person-years | 5063 | 1108 | 3955 |
| Number of events (%) | 141 (10%) | 38 (12%) | 103 (9%) |
| Events per 1,000 patient years | 27.9 | 34.3 | 26.0 |
